# Complete Genome Sequence of Staphylococcus epidermidis CCSM0287, Isolated from Healthy Facial Skin

**DOI:** 10.1128/mra.00131-23

**Published:** 2023-03-20

**Authors:** Laiji Ma, Suzhen Yang, Hong Jiang, Chunying Yuan, Huidan Chen, Yan Li, Li Shao

**Affiliations:** a School of Perfume and Aroma Technology, Shanghai Institute of Technology, Shanghai, People’s Republic of China; b R&D Innovation Center, Shandong Freda Biotech Co., Ltd., Jinan, Shandong, People’s Republic of China; University of Rochester School of Medicine and Dentistry

## Abstract

Staphylococcus epidermidis strain CCSM0287 was isolated from healthy facial skin. The complete genome of CCSM0287 was sequenced using a combination of Pacific Biosciences (PacBio) RS II single-molecule real-time (SMRT) and Illumina sequencing. The assembled 2.5-Mbp genome consisted of one chromosome and three plasmids.

## ANNOUNCEMENT

Staphylococcus epidermidis is a ubiquitous inhabitant of human skin and plays a crucial role in maintaining skin homeostasis. Various studies have shown that S. epidermidis is the guardian of skin health, promoting skin immunity, repair, and antimicrobial defense (1–8). S. epidermidis CCSM0287 was isolated from the healthy facial skin of a Chinese woman.

In 2021, a skin microbial sample was collected from the cheek of a healthy woman in Shanghai, China (ethics approval number 2021-KY-15: Shanghai Jiaotong University Affiliated Sixth People’s Hospital South Campus), with a sterile cotton swab and sterile collection solution containing 0.9% NaCl and 0.1% Tween 20. The sample was mixed, serially diluted with sterile water, and then cultured on a tryptic soy agar (TSA) plate containing 5% defibrated sheep blood. After anaerobic incubation at 37°C for 12 to 16 h, small, white, round colonies were picked out and then restreaked at least three times on TSA plates to obtain pure cultures; 16S rRNA gene sequencing was carried out using the universal primers 27F and 1492R.

Strain CCSM0287 was recovered from frozen storage on a TSA plate and then cultured aerobically in tryptic soy broth at 37°C for 12 to 16 h with shaking. Genomic DNA was extracted from CCSM0287 using the Wizard genomic DNA purification kit (Promega). Purified genomic DNA was quantified with a TBS-380 fluorometer (Turner BioSystems Inc., Sunnyvale, CA) and then sequenced using a combination of Pacific Biosciences (PacBio) RS II single-molecule real-time (SMRT) and Illumina sequencing platforms.

For Illumina sequencing, at least 1 μg genomic DNA was used for each strain for sequencing library construction. DNA samples were sheared into 400- to 500-bp fragments with a Covaris M220 focused acoustic shearer following the manufacturer’s protocol. Illumina sequencing libraries were prepared from the sheared fragments using the NEXTFLEX rapid DNA-sequencing kit, Paired-end sequencing libraries (2 × 150 bp) were prepared using a NovaSeq 6000 S4 reagent kit v1.5 (300 cycles) and then were sequenced on an Illumina NovaSeq 6000 system. Raw data from sequencing were stored in fastq format, and fastp v0.23.0 (9) was used to trim raw data according to quality, which yielded 1,146,102,382 bp of raw bases, with 150-bp read length; clean reads with scores of Q20 accounted for 97.69% of all reads. For PacBio sequencing, an aliquot of 15 μg DNA was centrifuged in a g-TUBE (Covaris, MA) at 6,000 rpm for 60 s using an Eppendorf 5424 centrifuge, which processed genomic DNA into ~10-kb fragments. DNA fragments were then purified, end repaired, and ligated with SMRTbell sequencing adapters following the manufacturer’s recommendations (PacBio, San Diego, CA), which yielded 361,100 reads with an average length of 10,966.19 bases and an *N*_50_ value of 11,956 bp.

The Illumina and PacBio read data were assembled *de novo* using Unicycler v0.4.8 (10) to yield structurally consistent assembled sequences, which were then iteratively polished with Pilon v1.22 (11) to generate a single circular chromosome of 2,458,918 bp (GC content, 32.11%) and three circular plasmids, i.e., plasmid A (58,549 bp), plasmid B (6,823 bp), and plasmid C (6,796 bp) (Table 1). Rotation and circularity were confirmed via Unicycler. Glimmer v3.02 (12) was used for coding sequence (CDS) prediction, tRNAscan-SE v2.0 (13) was used for tRNA prediction, and Barrnap v0.9 (14) was used for rRNA prediction; 2,332 CDSs, 19 rRNAs, 60 tRNAs, and 83 small RNAs were identified. Default parameters were used for all software unless otherwise specified.

**TABLE 1:** Genome statistics and features of Staphylococcus epidermidis strain CCSM0287

Chromosome or plasmid	Length (bp)	GC content (%)	No. of CDSs	No. of rRNAs	No. of tRNAs	GenBank accession no.
Chromosome	2,458,918	32.11	2,332	19	60	CP110611
Plasmid A	58,549	27.96	60	0	0	CP110612
Plasmid B	6,823	31.64	9	0	0	CP110613
Plasmid C	6,796	30.19	11	0	0	CP110614

### Data availability.

The complete genome sequence of CCSM0287 is available in GenBank under accession numbers CP110611 to CP110614, BioProject accession number PRJNA893857, BioSample accession number SAMN31433203, and Sequence Read Archive (SRA) accession number SRP422170. S. epidermidis strain CCSM0287 has been deposited in the China Center for Type Culture Collection under accession number CCTCC M2022779.
